# The Brassicaceae-Specific *EWR1* Gene Provides Resistance to Vascular Wilt Pathogens

**DOI:** 10.1371/journal.pone.0088230

**Published:** 2014-02-05

**Authors:** Koste A. Yadeta, Dirk-Jan Valkenburg, Mathieu Hanemian, Yves Marco, Bart P. H. J. Thomma

**Affiliations:** 1 Laboratory of Phytopathology, Wageningen University, Wageningen, The Netherlands; 2 Laboratoire des Interactions Plantes Microorganismes, Centre National de la Recherche Scientifique-Institut National de la Recherche Agronomique, Castanet-Tolosan, France; Ghent University, Belgium

## Abstract

Soil-borne vascular wilt diseases caused by *Verticillium* spp. are among the most destructive diseases worldwide in a wide range of plant species. The most effective means of controlling Verticillium wilt diseases is the use of genetic resistance. We have previously reported the identification of four activation-tagged Arabidopsis mutants which showed enhanced resistance to Verticillium wilt. Among these, one mutant also showed enhanced resistance to *Ralstonia solanacearum*, a bacterial vascular wilt pathogen. Cloning of the activation tag revealed an insertion upstream of gene *At3g13437*, which we designated as *EWR1* (for *Enhancer of vascular Wilt Resistance 1*) that encodes a putatively secreted protein of unknown function. The search for homologs of Arabidopsis *EWR1* (*AtEWR1*) in public databases only identified homologs within the *Brassicaceae* family. We subsequently cloned the *EWR1* homolog from *Brassica oleracea* (*BoEWR1*) and show that over-expression in Arabidopsis results in *V. dahliae* resistance. Moreover, over-expression of *AtEWR1* and *BoEWR1* in *N. benthamiana*, a member of the Solanaceae family, results in *V. dahliae* resistance, suggesting that *EWR1* homologs can be used to engineer Verticillium wilt resistance in non-Brassicaceae crops as well.

## Introduction

*Verticillium* species belong to the phylum Ascomycota that comprises the largest group of fungal pathogens and contains several plant pathogenic species such as *V. dahliae* and *V. longisporum* [1], [2], [3]. While *V. dahliae* has an extremely broad host range that contains hundreds of mainly dicotyledonous plant hosts, *V. longisporum* is pathogenic on *Brassicaceae* only. *Verticillium* spp. are soil-borne and cause vascular wilt diseases [1], [2], [4]. Controlling Verticillium wilt diseases is difficult for several reasons: *Verticillium* spp. produce resting structures that can survive in the soil for many years [5], and soil fumigation has largely been banned due to environmental concerns. A commonly used alternative control method, crop rotation, is ineffective due to the wide host range of the pathogen. Finally, once into the xylem, the fungus is not affected by fungicides. Consequently, the preferred method to control Verticillium wilt disease is the use of genetic resistance.

Genetic resistance against Verticillium wilt diseases has been reported for several crop species [1], [6]. The first Verticillium wilt resistance locus that has been cloned and functionally characterized is the tomato *Ve* locus that contains the *Ve1* gene that provides resistance in tomato against race 1 isolates of *V. dahliae* [7], [8]. Recently, it was shown that transgenic expression of tomato *Ve1* in Arabidopsis provides resistance against *Verticillium* race 1 isolates [9]. Over the years, Arabidopsis has increasingly been used as a model host to study *Verticillium*-host interactions [1], [10], [11], [12], [13], [14]. In addition to screening germplasm for resistance [12], [15], mutagenesis followed by screening for enhanced resistance with a pathogen of interest is a means to identify novel resistance traits. Several molecular techniques have been used to generate random mutants in Arabidopsis, such as EMS- and radiation-induced mutation, and transposon and activation tagging. Activation tagging involves the random integration of promoter or enhancer sequences in a genome, using either a T-DNA or a transposon, generally leading to enhanced expression of genes near the integration site and generating gain-of-function mutations [16], [17], [18]. Insertion of enhancer sequences in the genome may positively regulate gene expression, even when inserted at a considerable distance to the target gene [19]. Some of the advantages of activation tagging over knock-out strategies include that activation tagging generates dominant instead of recessive mutations, it generates viable mutants for those genes where knock-outs would lead to lethal phenotypes and it is also applicable to dissect phenotypes of redundant genes [18].

Transposon-based activation tagging has been successfully employed in various plant species to identify novel genes involved in various physiological processes [17], including pathogen defence [20], [21], [22]. In an attempt to identify sources of Verticillium wilt resistance using Arabidopsis, we have screened an activation-tagged Arabidopsis mutant collection with *V. dahliae*. Previously, we have reported the identification of four mutants with enhanced resistance to Verticillium wilt disease [23]. Here, we pursued functional characterization of one of the mutants and demonstrate that enhanced activation of the *At3g13437* gene, encoding a protein of unknown function, is responsible for the enhanced Verticillium wilt resistance phenotype. This gene is designated as *Enhancer of vascular Wilt Resistance 1* (*EWR1*).

## Results

### Identification of the *Enhancer of vascular Wilt Resistance 1*

**Figure 1:**
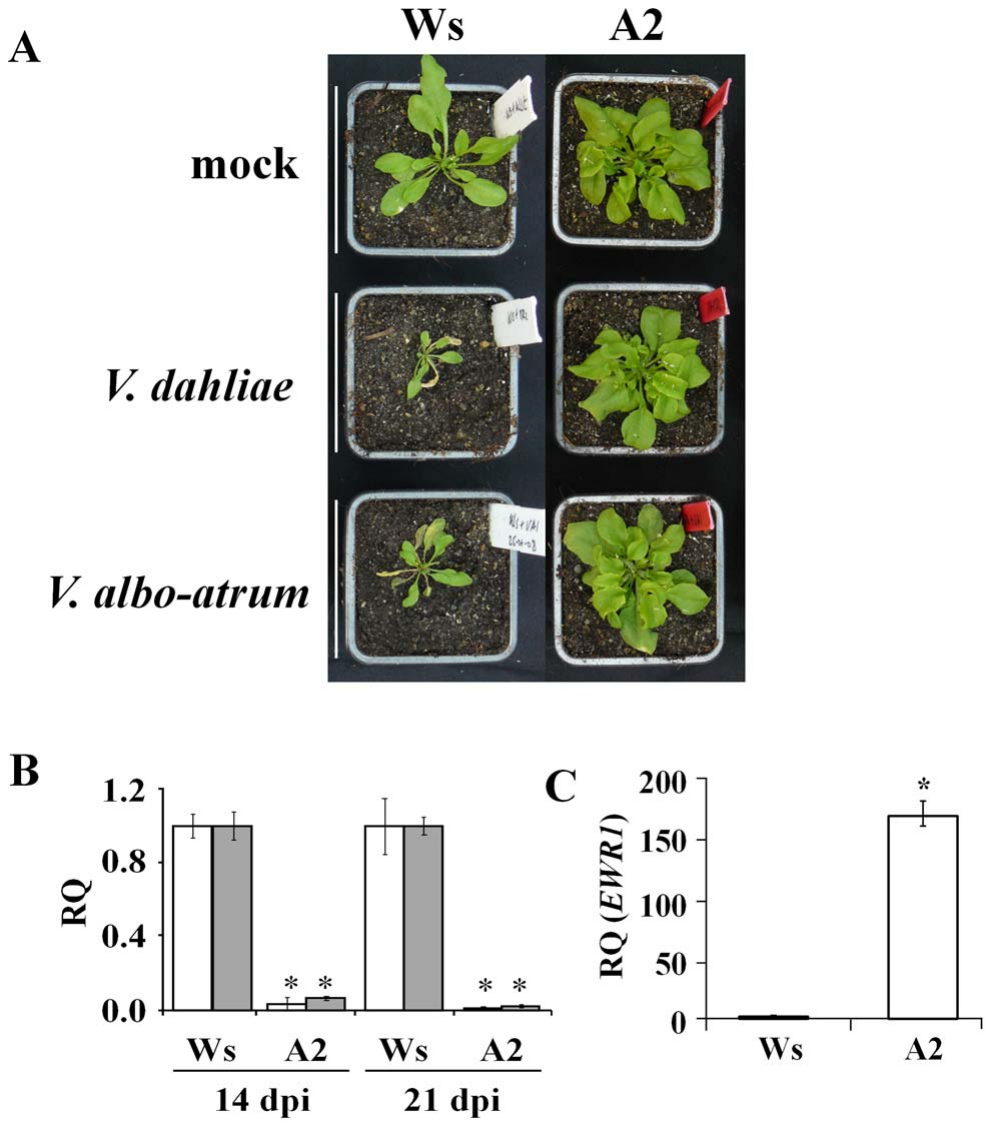
The activation-tagged Arabidopsis mutant A2 is more resistant to *V. dahliae* and *V. albo-atrum*. (A) Typical symptoms of *Verticillium* on the wild-type (WS) and the activation-tagged mutant A2. Picture was taken at 21 days post inoculation (dpi) and a representative of three independent experimental replicates is shown. (B) Relative quantification (RQ) by real-time PCR of Verticillium colonization by comparing levels of the *V. dahliae* (white bars) and *V. albo-atrum* (grey bars) internal transcribed spacer (ITS) region of the ribosomal DNA (as measure for fungal biomass) relative to levels of the large subunit of the Arabidopsis *RubisCo* gene (for equilibration) at 14 and 21 dpi. Bars represent averages with standard deviation of four technical replicates. A representative of three independent experiments is shown. (C) Relative quantification (RQ) of *EWR1* transcription level in the wild-type WS and the activation-tagged mutant A2. The bar represents the average of three independent experiments and standard deviation of the means and asterisks indicate significant differences (Dunnett t-test at *P = 0.01*) compared to the wild-type WS.

**Figure 2:**
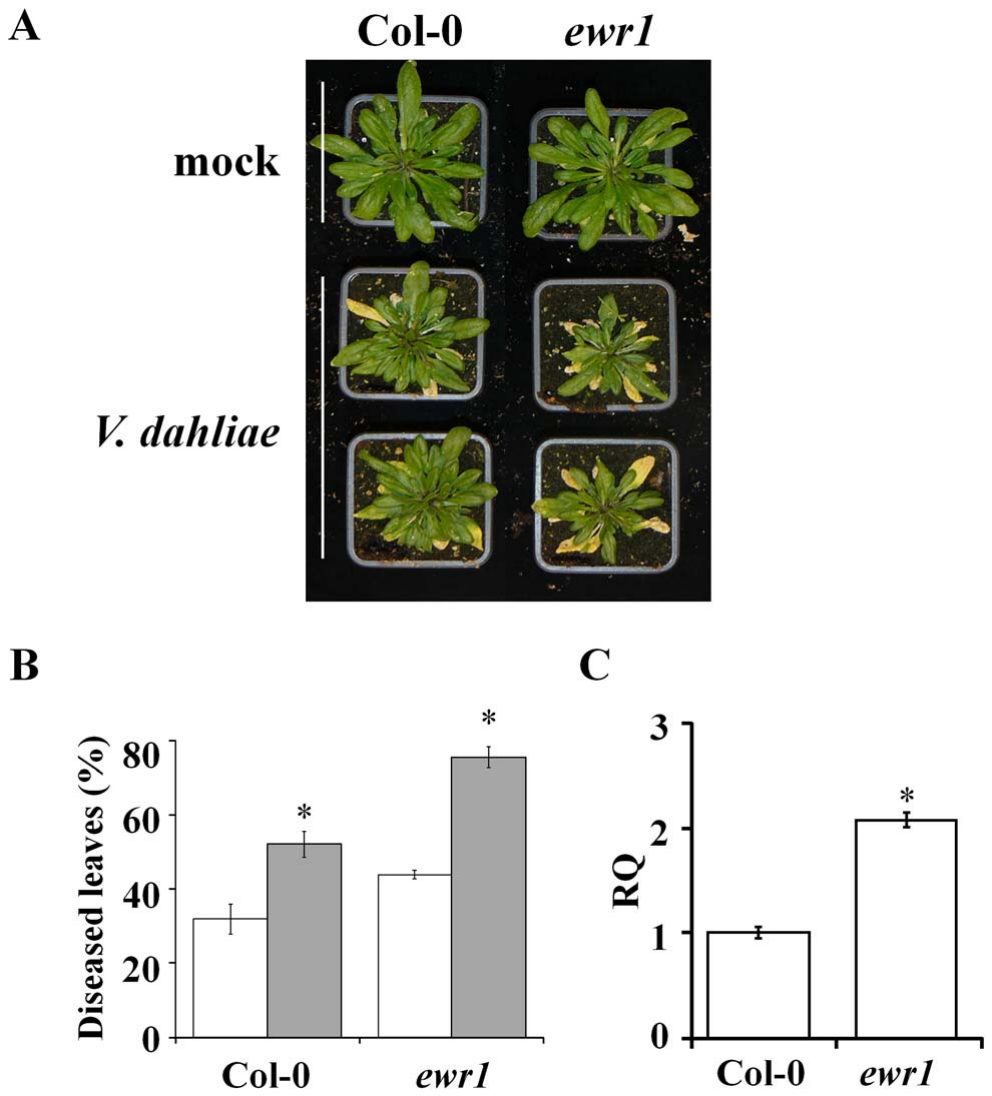
Deletion of *EWR1* enhances Arabidopsis susceptibility to Verticillium wilt. (A) Typical symptoms of *V. dahliae* on the wild-type (Col-0) and *EWR1* knock out (*ewr1*) plants. Picture was taken at 21 days post inoculation (dpi) and a representative of three independent experimental replicates is shown. (B) Disease severity score for the wild-type (Col-0) and *ewr1* at 14 (white bar) and 21 (grey bar) days post inoculation (dpi). The total number of rosette leaves and the number of rosette leaves that showed Verticillium symptoms were counted at least from eight plants and percentage of the diseased leaves were calculated as an indication of disease severity. The bars represent averages of three independent experiments with standard deviation and asterisks indicate significance differences (Dunnett t-test at *P = 0.05*). (C) Relative quantification (RQ) by real-time PCR of Verticillium colonization by comparing levels of the *V. dahliae* internal transcribed spacer (ITS) region of the ribosomal DNA (as measure for fungal biomass) relative to levels of the large subunit of the Arabidopsis *RubisCo* gene (for equilibration) at 21 dpi. Bars represent averages with standard deviation of four technical replicates. A representative of three independent experiments is shown.

Previously, we have reported the identification of four activation-tagged Arabidopsis mutants, A1 to A4, that displayed enhanced resistance to Verticillium wilt disease [23]. Of these, mutant A2 not only displayed resistance to *V. dahliae* (Figure 1), but also to the bacterial vascular wilt pathogen *Ralstonia solanacearum* [23]. Here, we investigated this mutant further and determined the position of the activation tag insertion site using thermal asymmetric interlaced PCR (TAIL-PCR) [24]. The tag was found to be inserted on chromosome 3 at a position 376 bp upstream of the translational start codon of gene *At3g13435*. To identify the gene responsible for the enhanced Verticillium resistance of the A2 mutant, we analysed the expression of genes flanking the T-DNA insertion site to detect transcriptional changes. The analysis of expression of 11 genes spanning a region of ∼14 kb upstream to ∼17 kb downstream of the activation tag insertion site showed that four of these genes, namely *At3g13432*, *At3g13435, At3g13437* and *At3g13445*, were induced in the A2 mutant when compared to wild-type plants (Table 1, Figure S1 in File S1). Simultaneously, we analysed homozygous knock-out alleles of all genes flanking the activation tag insertion site for susceptibility towards *V. dahliae*. Interestingly, the knock-out allele of *At3g13437* showed clearly enhanced susceptibility to *V. dahliae* (Figure 2A), whereas none of the other knock-out alleles showed enhanced susceptibility to Verticillium wilt when compared to the Col-0 wild-type (Table 1). Therefore, we tentatively named *At3g13437 EWR1*, for *Enhancer of vascular Wilt Resistance 1*. To validate the enhanced susceptibility of the knock-out allele of *At3g13437*, *ewr1*, we quantified the ratio of rosette leaves showing Verticillium wilt symptoms at 14 and 20 days post inoculation (dpi), showing that the percentage of diseased rosette leaves of the *ewr1* mutant is significantly higher when compared with wild-type plants (Figure 2B). We further validated the enhanced susceptibility of *ewr1* by quantifying *V. dahliae* colonization of the rosette leaves using real-time PCR. As expected, more fungal DNA was detected in *ewr1* plants when compared with wild-type plants (Figure 2C). Overall, the gene expression data, combined with the Verticillium wilt phenotyping, strongly suggests that enhanced expression of *EWR1* causes enhanced Verticillium wilt resistance in the activation-tagged mutant A2.

**Table 1:** Analysis of the genes flanking the activation-tag insertion site in mutant A2.

Gene	Annotation	Knock-out allele	Expression^1^	*Verticillium* phenotype^2^
*At3g13405/03*	MicroRNA	SALK_113174C	Not tested	Similar
*At3g13410*	Unknown protein	Unavailable	Similar	Not tested
*At3g13420*	Zinc finger family	SALK_041147C	Similar	Similar
*At3g13430*	Zinc finger family	SALK_135697	Similar	Similar
*At3g13432*	Unknown protein	Unavailable	Induced in A2 mutant	not tested
*At3g13435*	Unknown protein	SALK_091102	Induced in A2 mutant	Similar
***At3g13437***	**Unknown protein**	**SALK_139498C**	**Induced in A2 mutant**	**Enhanced susceptibility**
*At3g13440*	Methyltransferase/nucleic acid binding protein	SALK_020621	Similar	Similar
*At3g13445*	TATA binding protein	SALK_084279C	Induced in A2 mutant	Similar
*At3g13450*	Alpha-keto acid dehydrogenase E1	SALK_042796C	Similar	Similar
*At3g13460*	ECT2 like (Physically interacts with CIPK1)	SALK_002225C	Similar	Similar

1 Gene expression in mutant A2 relative to the expression in wild-type.

2 Phenotype of knock-out alleles upon *V. dahliae* inoculation when compared to wild-type plants.

We have previously shown that the induction of *AHL19*, which encodes an AT-hook DNA binding protein, is causal to the enhanced Verticillium wilt resistance in the A1 mutant [23]. To investigate whether *EWR1* over-expression can explain the enhanced Verticillium wilt resistance of the A3 and A4 mutants, we assessed *EWR1* expression in these mutants in absence of pathogen challenge. This analysis showed that *EWR1* is not over-expressed in these mutants (Figure S2 in File S1), showing that constitutive activation of *EWR1* cannot explain the enhanced Verticillium wilt resistance in the A3 and A4 mutants.

### *EWR1* over-expression provides resistance to Verticillium wilt

**Figure 3:**
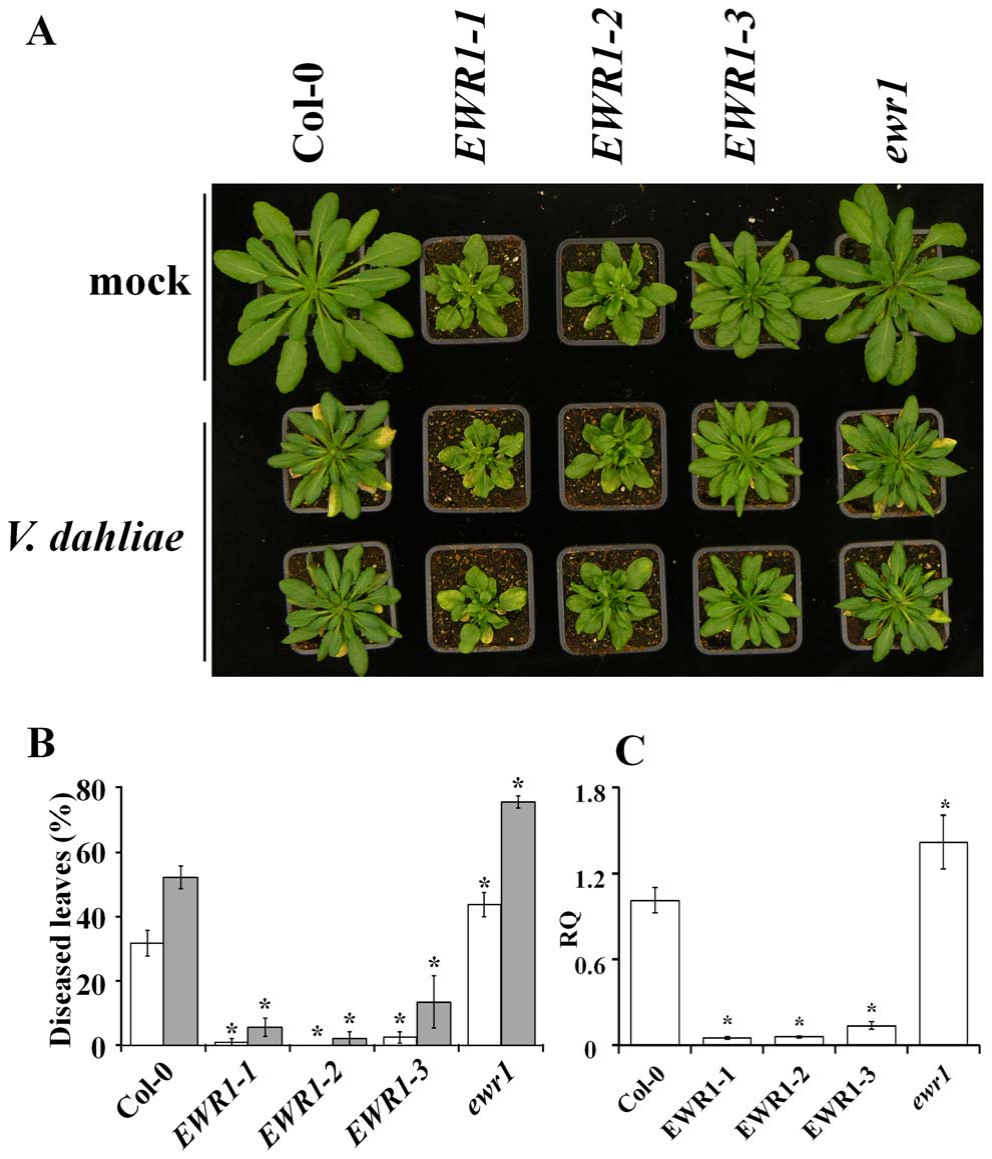
*EWR1* over-expressing Arabidopsis plants are resistant to *V. dahliae*. (A) Typical symptoms of *V. dahliae* on the wild-type (Col-0), three *EWR1* expressing lines (EWR1-1, EWR1-2, and EWR1-3) and *EWR1* knock out line (*ewr1*). Picture was taken at 21 days post inoculation and a representative of three experimental replicates is shown. (B) Disease severity score for the wild-type (Col-0), the three *EWR1* expressing lines (EWR1-1, EWR1-2, and EWR1-3) and *EWR1* knock out line (*ewr1*) at 14 (white bar) and 21 (grey bar) days post inoculation (dpi). The total number of rosette leaves and the number of rosette leaves that showed Verticillium symptoms were counted at least from eight plants and percentage of the diseased leaves were calculated as an indication of disease severity. The bars represent the average of three independent experiments with standard deviation and asterisks indicate significance differences (Dunnett t-test at *P = 0.05*). (C) Relative quantification (RQ) by real-time PCR of Verticillium colonization by comparing levels of the *V. dahliae* internal transcribed spacer (ITS) region of the ribosomal DNA (as measure for fungal biomass) relative to levels of the large subunit of the Arabidopsis *RubisCo* gene (for equilibration) at 21 dpi. Bars represent averages with standard deviation of four technical replicates. A representative of three independent experiments is shown.

To corroborate whether the enhanced expression of *EWR1* is causal to the enhanced Verticillium wilt resistance of mutant A2, we generated *EWR1* over-expressing lines in Arabidopsis ecotypes Col-0 and WS. Similar to the activation-tagged mutant A2, which displays compact and rounded rosette leaves with short petioles [23], also *EWR1* over-expressing plants displayed altered plant morphology (Figure 3A, Figure S3A in File S1). *EWR1* over-expressing plants show compact, dark green and slightly thicker leaves than wild-type plants with short petioles, and shorter and fewer inflorescences (Figure S4 in File S1).

In order to learn if *EWR1* over-expression contributes to Verticillium wilt resistance, three independent *EWR1* over-expressing lines of the Col-0 ecotype (*EWR1*-*1*, *EWR1*-*2*, and *EWR1*-*3*) were challenged with *V. dahliae* along with *ewr1* and wild-type plants. While Col-0 and *ewr1* plants showed clear wilting, chlorosis, and stunting symptoms at 14 dpi that were significantly increased by 21 dpi, *EWR1*-expressing plants showed only mild symptoms at these time points (Figure 3A; Figure S3A in File S1). Thus, when assessing the impact of *V. dahliae* inoculation by comparing mock-inoculated and *V. dahliae*-inoculated plants for each of the genotypes it is evident that *EWR1* over-expressing lines show relatively little impact of the pathogen. The degree of stunting induced by *V. dahliae* on wild-type plants (when comparing mock-inoculated and *V. dahliae*-inoculated Col-0 plants) and on *ewr1* plants (when comparing mock-inoculated and *V. dahliae*-inoculated *ewr1* plants) exceeds the degree of stunting induced by *V. dahliae* on *EWR1* over-expressing lines (when comparing mock-inoculated and *V. dahliae*-inoculated *EWR1* plants) by far. Moreover, quantification of fungal colonization *in planta* using real-time PCR showed only little *V. dahliae* biomass in *EWR1*-transgenic lines (Figure 3C). Similar phenotypes were observed on *EWR1* over-expressing lines of the WS ecotype (Figure S3 in File S1). These data further confirm that the constitutive activation of *EWR1* expression is causal to the enhanced Verticillium resistance of the A2 mutant.

### Transcriptional regulation of *EWR1*

We showed that constitutive over-expression of *EWR1* enhances Arabidopsis resistance to Verticillium wilt disease (Figure 1A, 3A, Figure S3A in File S1). To understand how *EWR1* is regulated at transcriptional level during the course of the Verticillium-Arabidopsis interaction, we performed a time course experiment where we challenged the wild-type Col-0 and WS plants with *V. dahliae*. Subsequently, we assessed transcription of *EWR1* using real-time PCR. This analysis showed that *EWR1* expression is transiently induced upon *V. dahliae* inoculation in both Col-0 and WS ecotypes (Figure S5 in File S1). Subsequently, the expression level of *EWR1* was assessed in roots and shoots of non-inoculated WS, A2 mutant and *EWR1*-*5* mutant plants. Except for *EWR1-4*, which showed slight induction, *EWR1* expression was hardly detected in roots of WS and mutant A2 whereas in shoots, *EWR1* was strongly expressed in mutant A2 and *EWR1-4* when compared with wild-type plants (Figure S6 in File S1).

### *EWR1* provides resistance to other vascular wilt pathogens

**Figure 4:**
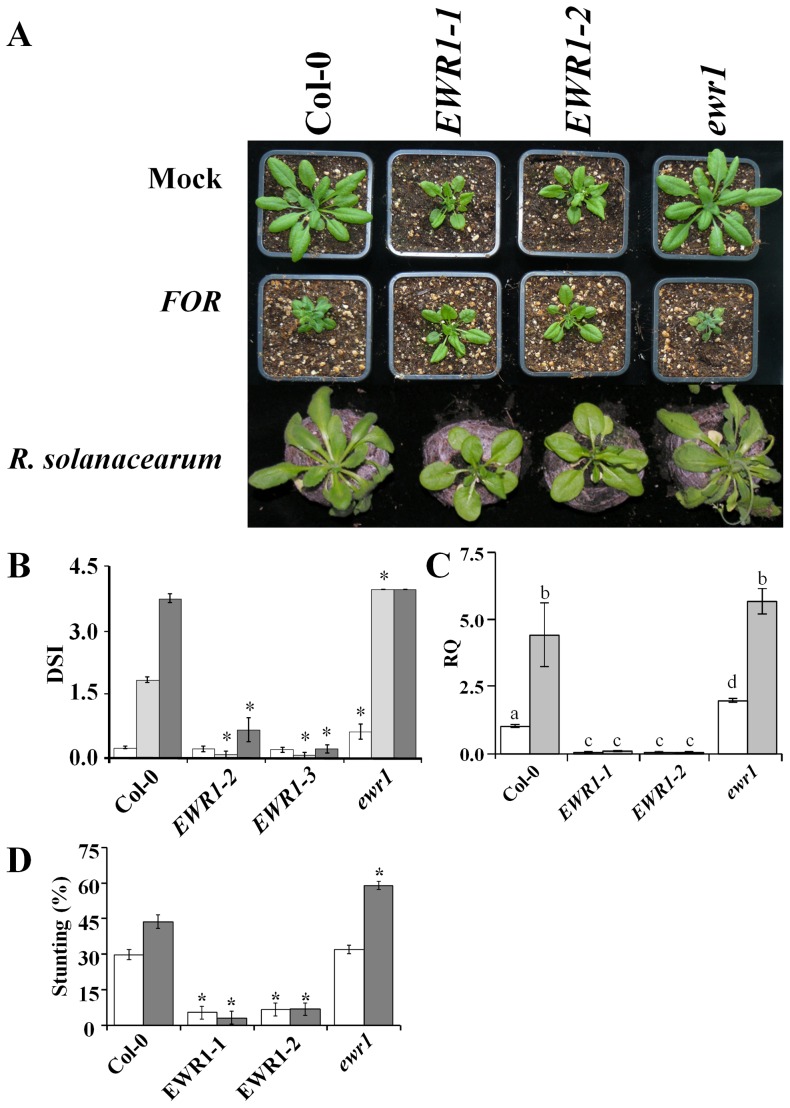
*EWR1* over-expression provides resistance to other vascular wilt pathogens. (A). typical disease symptoms caused by *Fusarium oxysporum* f.sp. *raphani* (*FOR*) and *R. solanacearum* on the wild-type (Col-0), two EWR1 expressing plants (EWR1-1 and EWR1-2) and the *EWR1* knock out line (*ewr1*) at 12 (*F. oxysporum*) and 5 (*R. solanacearum*) days post inoculation (dpi). The experiment was repeated at least three times and representative of the three replications is shown. (B) Disease severity index (DSI) scores upon inoculation of at least 21 plants with *R. solanacearum* on a scale of 0 (no infection) to 4 (all rosette leaves diseased) at 3 (white bar), 6 (light grey bar) and 10 (dark grey bar) dpi. Bars represent averages with standard deviation of three independent biological replicates and asterisks indicate significant differences (*p* = 0.05). (C) Relative quantification (RQ) by real-time PCR of *R. solanacearum* colonization in wild-type (Col-0), two independent *EWR1* over-expressing lines (EWR1-1 and, EWR1-2), and of the *EWR1* knockout line (*ewr1*) by comparing levels of the *R. solanacearum* endoglucanase gene (as measure for Ralstonia biomass) relative to levels of the large subunit of the Arabidopsis *RubisCo* gene (for equilibration) at 3 and 5 dpi. Bars represent averages with standard deviation of four technical replicates and a representative of three independent experiments is shown. (D) Fusarium-induced stunting of wild-type (Col-0) plants, two independent *EWR1* over-expression lines (EWR1-1 and, EWR1-2) and of the *EWR1* knockout line (*ewr1*) at 10 and 14 dpi. Rosette diameters of inoculated plants were compared with those of mock-inoculated plants. The bars represent averages of two independent experiments with standard deviation and asterisks indicate significant differences (Dunnett t-test at *P = 0.05*). (E) Relative quantification (RQ) of *EWR1* transcription in wild-type (Col-0) plants, two independent *EWR1* over-expressing plants (EWR1-1 and EWR1-2), the A2 mutant, and of the *EWR1* knock out line (*ewr1*). Bars represent averages with standard deviation of three biological replicates.

To investigate whether the enhanced *Ralstonia* resistance in the A2 mutant can similarly be attributed to *EWR1* over-expression, we challenged two *EWR1* over-expressing lines (*EWR1*-*1* and *EWR1*-*2*), along with the wild-type Col-0 and the *ewr1* mutant with *R. solanacearum* strain GMI1000. While Col-0 plants showed mild disease symptoms at 3 dpi which aggravated by 6 dpi (Figure 4 A, B), resulting in death of the inoculated plants by 10 dpi, most rosette leaves of GMI1000-inoculated *ewr1* plants showed clear wilting at 3 dpi and completely collapsed by 6 dpi, indicating that *ewr1* plants show enhanced susceptibility to *R. solanacearum*. Conversely, *EWR1* over-expressing plants were completely resistant to *R. solanacearum* and did not show any disease symptoms throughout the assay up to 10 dpi (Figure 4A, B). With real-time PCR it was confirmed that the amount of disease symptoms observed on the various genotypes correlates with the degree of *R. solanacearum* colonization (Figure 4C). Hardly any bacterial DNA was detected in DNA extracts of *EWR1*-*1* and *EWR1*-*2* rosette leaves, indicating that *EWR1* over-expression provides a high level of *R. solanacearum* resistance.

We have previously shown that the activation-tagged mutant A2 displayed wild-type levels of susceptibility to *Fusarium oxysporum* f. sp. *raphani* [23]. In this study, we have also challenged the *EWR1* over-expressing plants with *F. oxysporum*. While wild-type plants that were inoculated with *F. oxysporum* showed clear wilting of rosette leaves and overall stunting of the plants at 10 dpi which led to a complete collapse of the plants by 14 dpi, *EWR1* over-expressing plants showed enhanced resistance to this pathogen (Figure 4A, D). Inoculated *EWR1-1* and *EWR1-2* plants hardly showed any symptoms of disease throughout the assay. To explain the discrepancy in disease phenotypes between the A2 mutant and the *EWR1* over-expressing lines, we compared the *EWR1* expression levels in these plants, showing that *EWR1* expression is significantly higher in the over-expression lines when compared to the A2 mutant (Figure S2 in File S1).

### *EWR1* over-expression enhances drought tolerance

**Figure 5:**
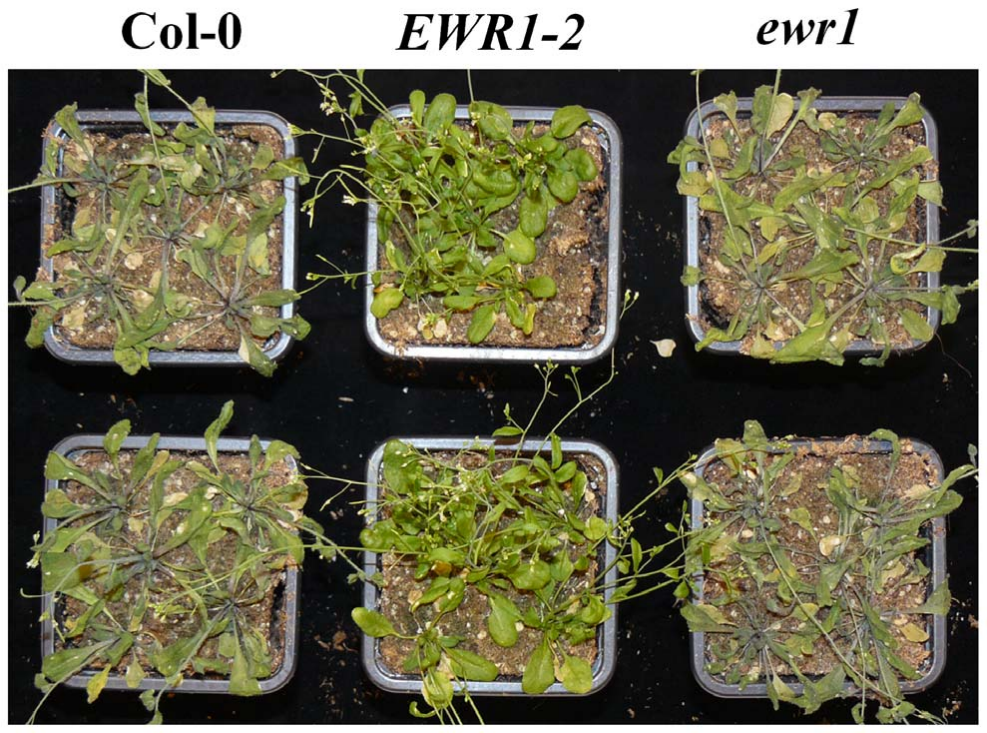
*AtEWR1* over-expressing plants are tolerant to drought stress. Three weeks-old wild-type Col-0, *AtEWR1* expressing line (EWR1-2) and *AtEWR1* knock out line (*ewr1*) plants were exposed to drought stress and picture was taken at 14 days post drought treatment. The assay was repeated three times and a representative of the replicates is shown.

In addition to enhanced Verticillium wilt resistance, we investigated whether over-expression of *EWR1* plays a role in drought stress tolerance. To this end, we tested the drought stress resistance of *EWR1*-*2*, the Col-0 wild-type and the *ewr1* mutant. After 3 weeks of growth with regular watering, we stopped watering the plants and evaluated the response of the lines to drought stress. The assay showed that Col-0 plants and *ewr1* mutants started to show wilting symptoms 10 days after the last watering, while the rosette leaves were collapsed after 14 days. In contrast, *EWR1* expressing plants did not show any drought symptoms up to 14 days after the last watering (Figure 5). We similarly evaluated the drought stress resistance of two *EWR1* over-expressing lines in WS background along with the WS wild-type and the activation-tagged mutant A2 and found similar results. *EWR1* over-expressing plants and A2 mutant showed enhanced drought tolerance when compared to the wild-type WS. This indicates that *EWR1* over-expression enhances drought stress tolerance in Arabidopsis.

### *EWR1* encodes a protein of unknown function

**Figure 6:**
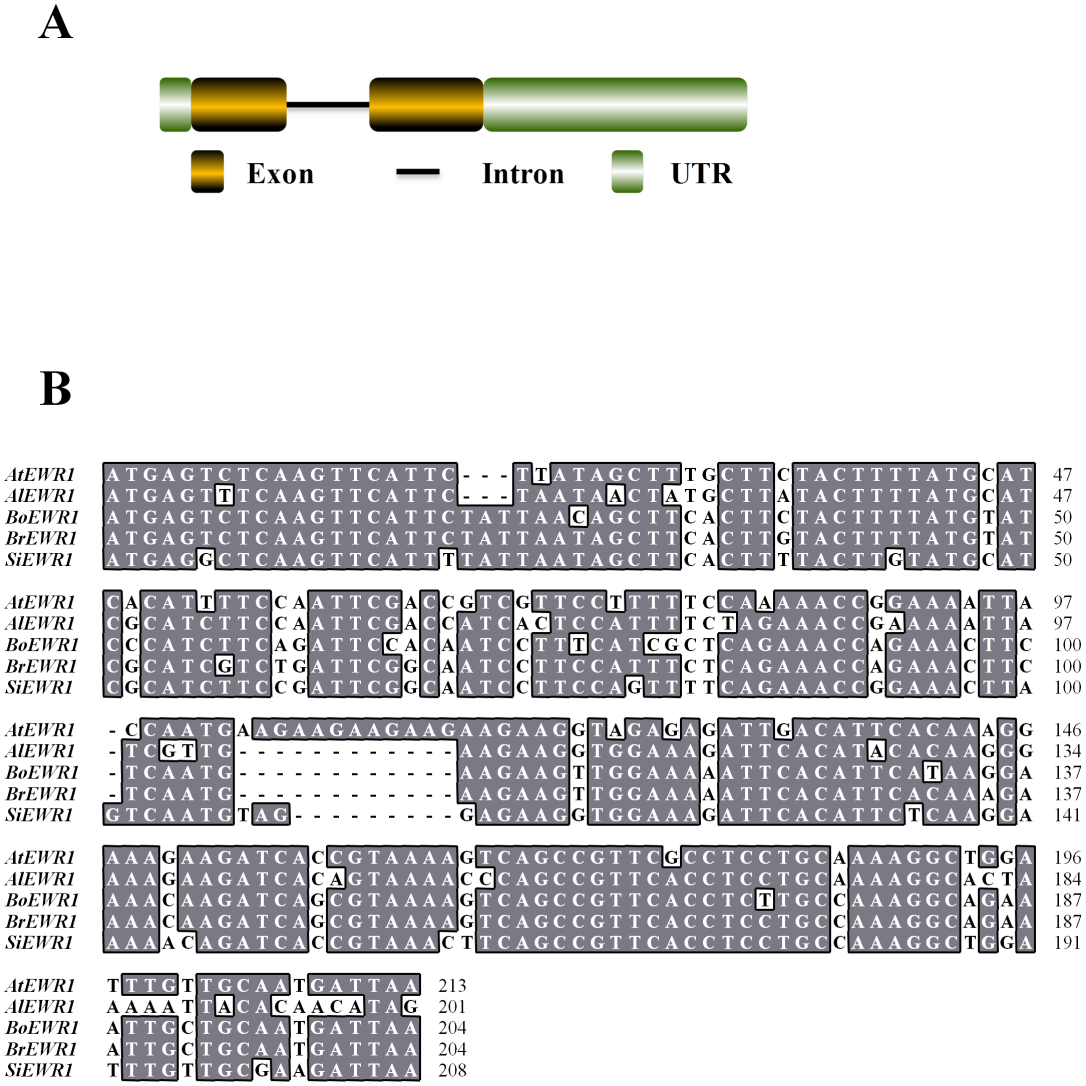
*EWR1* is highly conserved in *Brassicaceae*. (A) Schematic representation of the full-length genomic DNA sequence of *EWR1* gene. (B) Nucleotide sequence alignment of *AtEWR1* and its homologs from *Arabidopsis lyrata* (*AlEWR1*), *Brassica oleracea* var. *gemmifera* (*BoEWR1*), *Brassica rapa (BrEWR1)*, and *Sisymbrium irio* (*SiEWR1*).

The full-length genomic DNA sequence of *EWR1* consists of 599 bp containing two exons of 97 and 116 bp separated by an intron of 87 bp (Figure 6A). The ORF encodes a protein of 70 amino acids with a predicted molecular mass of 7.93 kDa which is annotated as unknown. With SignalP 3.0 (http://www.cbs.dtu.dk/services/SignalP/) [25] it is strongly predicted that EWR1 contains a signal peptide of 21 amino acids. Searching for recognizable protein domains or signatures of conserved motifs in the EWR1 protein sequence in publicly available databases did not result in any significant hits.

### A *B. oleracea EWR1* homolog provides Verticillium wilt resistance in Arabidopsis

A tblastx search using the nucleotide sequence of *EWR1* in the NCBI database (http://blast.ncbi.nlm.nih.gov/Blast.cgi) identified only three significant hits, all within the Brassicaceae family. The hits were with *Arabidopsis lyrata* (E-value = 1e-15), *Sisymbrium irio* clone SIR-40E09 (E-value = 4e-11) and *Brassica rapa* subsp. *pekinensis* clone KBrH040N18 (E-value = 9e-08) (Figure 6B). No *EWR1* homologs were identified in other plant species, suggesting that *EWR1* is a Brassicaceae-specific gene.

**Figure 7:**
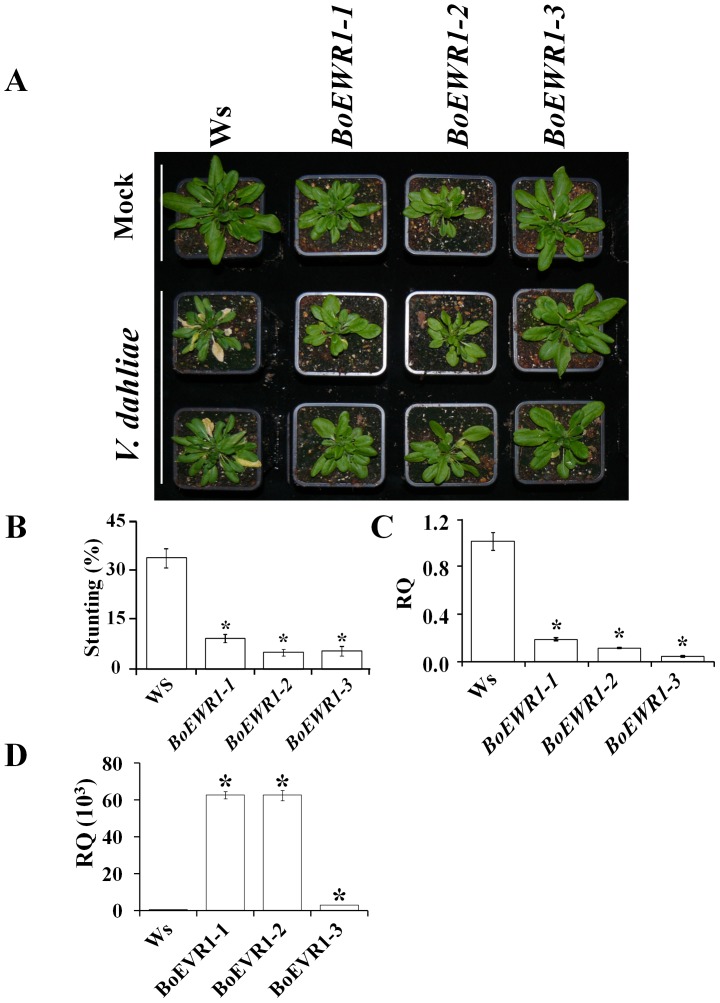
*BoEWR1* over-expression enhances Arabidopsis resistance to Verticillium wilt. (A). Typical disease symptoms caused by *V. dahliae* on the wild-type (WS) and three independent *BoEWR1* over-expressing plants (BoEWR1-1, BoEWR1-2 and BoEWR1-3) at 21 days post inoculation (dpi). The experiment was repeated at least three times and representative of the three independent biological replications is shown. (B) *Verticillium*-induced stunting of wild-type (WS), three independent *BoEWR1* over-expressing plants (BoEWR1-1, BoEWR1-2 and BoEWR1-3) at 21 dpi. Rosette diameters of inoculated plants were compared with those of mock-inoculated plants. The bars represent averages of three independent experiments with standard deviation and asterisks indicate significant differences (Dunnett t-test at *P = 0.05*). (C) Relative quantification (RQ) by real-time PCR of *Verticillium* colonization by comparing levels of the *V. dahliae* internal transcribed spacer (ITS) region of the ribosomal DNA (as measure for fungal biomass) relative to levels of the large subunit of the Arabidopsis *RubisCo* gene (for equilibration) at 21 dpi. Bars represent averages with standard deviation of four technical replicates. A representative of three independent experiments is shown. (D) Relative quantification (RQ) of *EWR1* transcription in wild-type (WS) and three independent *BoEWR1* over-expressing plants (BoEWR1-1 and BoEWR1-2, and BoEWR1-3). Bars represent averages with standard deviation of three biological replicates

Based on the sequence conservation, we designed primers to amplify the *EWR1* homologue from yet another Brassicaceous species, *Brassica oleracea*, using genomic DNA. Indeed a homolog, designated *BoEWR1*, was amplified which reinforced the suggestion that *EWR1* is a Brassicaceae-specific gene. We next aimed to test whether *BoEWR1* expression in Arabidopsis also provides Verticillium wilt resistance. To this end, *BoEWR1* was amplified from *B. oleracea* cDNA and constitutively expressed in the Arabidopsis Col-0 and WS ecotypes. As expected, *BoEWR1* expressing plants displayed similar leaf morphology as *AtEWR1* expressing plants (Figure 7A). Subsequently, *BoEWR1* expressing plants were challenged with *V. dahliae*, revealing enhanced resistance to Verticillium wilt when compared with wild-type plants (Figure 7A, B). Real-time PCR analysis confirmed reduced Verticillium colonization on *BoEWR1* expressing plants when compared with wild-type plants (Figure 7C). These data show that the *EWR1* homologs of *Arabidopsis thaliana* (*AtEWR1*) and *Brassica oleracea* (*BoEWR1*) are functional homologs with respect to their role in Verticillium wilt resistance.

### *AtEWR1* and *BoEWR1* over-expression in *N. benthamiana* confers Verticillium wilt resistance

**Figure 8:**
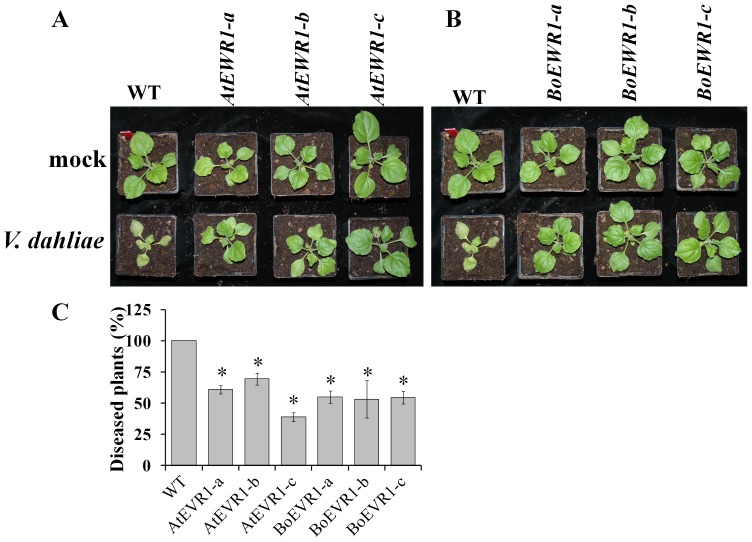
*AtEWR1* and *BoEWR1* over-expression in *N. benthamiana* results in resistance to *V. dahliae*. Typical symptoms of *V. dahliae* on the wild-type (WT), *AtEWR1* (*AtEWR1*-*a, b, c*) (A) and *BoEWR1* (*BoEWR1*-*a, b, c*) (B) over-expressing *N. benthamiana* plants. Pictures were made at 10 dpi and the upper and lower rows indicate mock- and *V. dahliae*-inoculated plants, respectively. (C) Percentage of plants (n = 20) that showed clear Verticillium symptoms at 10 dpi. Bars represent averages of three biological replications with standard deviations, and asterisks indicate significance differences when compared with WT (*P* = 0.01).

To investigate whether *AtEWR1* over-expression results in Verticillium wilt resistance in non-Brassicaceae plant species, we over-expressed *AtEWR1* and *BoEWR1* in the Australian tobacco species *Nicotiana benthamiana*. This Solanaceous plant species has been used as a model system to study interactions with various plant pathogens [26]. Unlike in Arabidopsis, *AtEWR1* or *BoEWR1* over-expression did not cause any obvious changes in the morphology of *N. benthamiana* plants when compared to non-transgenic control plants, except from perhaps slight stunting (Figure 8A, B). Based on the expression level of *EWR1* (Figure S7 in File S1), three independent *AtEWR1* (*AtEWR1*-a, *AtEWR1-b*, and *AtEWR1-c*) and *BoEWR1* (*BoEWR1-a*, *BoEWR1-b*, and *BoEWR1-c*) expressing T2 lines were selected and challenged with *V. dahliae*. Interestingly, *AtEWR1* and *BoEWR1* over-expressing *N. benthamiana* plants showed reduced Verticillium wilt symptoms when compared with inoculated wild-type plants (Figure 8A; B). The wild-type plants showed severe wilting, stunting, and chlorosis symptoms at 7 dpi, and leaves are completely collapsed by 10 dpi, whereas *AtEWR1* as well as *BoEWR1* over-expressing plants showed only mild wilting symptoms only on the older, lower leaves at 10 dpi (Figure 8A; B). When we counted the number of plants (n = 10 plants per assay) that showed any symptoms of Verticillium wilt disease at 10 dpi, irrespective of the severity of the symptoms, 100% of the wild-type plants showed signs of infection, whereas only 40 to 60% of the *AtEWR1* and *BoEWR1* over-expressing plants displayed symptoms (Figure 8C). This indicates that *EWR1* homologs from Brassicaceae species can be used to establish Verticillium wilt resistance in non-Brassicaceae plant species in the absence of significant developmental phenotypes.

## Discussion

Arabidopsis has increasingly been used as a model host for the identification of Verticillium resistance sources and studying the molecular mechanisms of *Verticillium*-host interactions [9], [10], [11], [12, [13], [14], [27]. In a phenotypic screening for gain-of-function mutants, we have previously reported the identification of four activation-tagged Arabidopsis mutants (A1–A4) which showed enhanced Verticillium wilt resistance [23]. We have also reported that the specific activation of the gene encoding the AT-hook DNA binding protein AHL19 causes enhanced Verticillium resistance in the A1 mutant [23]. Here, we cloned the activation-tag insertion site in the A2 mutant that showed enhanced resistance not only to *Verticillium* spp., but also to *R. solanacearum*. Among the 11 genes found within a 31 Kb window spanning the activation tag insertion site, four genes showed induced expression in the A2 mutant, of which *At3g13437* (*EWR1*) showed the strongest over-expression when compared to wild-type plants. Intriguingly, only the KO allele of *EWR1* showed increased Verticillium susceptibility when compared to wild-type, whereas over-expression of *EWR1* in wild-type Arabidopsis provided resistance to *V. dahliae*, *F. oxysporum* and *R. solanacearum*. Interestingly, in addition to enhanced resistance towards the *V. dahliae*, *V. albo-atrum* and *V. longisporum*, mutant A2 shows wild-type susceptibility to the necrotrophic foliar pathogens *B. cinerea*, and *P. cucumerina* and to the bacterial foliar pathogen *P. syringae* [23]. This suggests that *EWR1* over-expression does not lead to overall enhanced plant defence against a wide range of pathogens.

Soil-borne vascular wilt pathogens share several important features with respect to their biology and infection style. The pathogens enter their hosts via the roots, invade xylem vessels and spread rapidly to the aerial part of the plants [1], [2], [4], [28]. Thus, any plant resistance mechanism that prevents root penetration, xylem colonization or systemic spread could potentially contribute to resistance towards vascular wilt pathogens. We previously showed that the A2 mutant has similar root morphology as wild-type plants [23], suggesting that *EWR1*-mediated pathogen resistance is unlikely to be caused due to altered root morphology. Moreover, the stronger *EWR1* induction in shoots than in roots of the A2 mutant suggests that *EWR1*-mediated resistance may occur in shoots rather than in roots. Previous studies in Arabidopsis as well as in tomato have also shown that Verticillium wilt resistance is established once the fungus has entered and colonized the xylem vessels [8], [9], [23], [28], [29]. Despite absence of a significant difference in root morphology between the A2 mutant and the wild type plants, a significant morphological alteration in the rosette of the A2 mutant, which was even stronger in the rosette of *EWR1* over-expressing lines, was observed. The severity of the developmental phenotypes correlates with the level of *AtEWR1* expression that is significantly higher in the *AtEWR1* over-expression lines than in the A2 mutant (Figure S2 in File S1). However, irrespective of the extent of phenotypical deviations, all *EWR1*-expressing plants were found to display resistance that is specific to vascular wilt pathogens and does not concern other (foliar) pathogens. Furthermore, the developmental phenotypes that are observed upon *EWR1* over-expression are not observed upon over-expression in *N. benthamiana*, although vascular wilt resistance was maintained in these plants. Finally, all assays were performed in well-watered plants that did not experience drought stress, and no macroscopically visible signs of stress, such as anthocyanin accumulation, were observed. These findings suggest that the induction of developmental aberrancies and Verticillium wilt resistance can be uncoupled.

*EWR1* encodes a mature protein of 49 amino acids with unknown function. Homologs are only found in *Brassicaceae* species, showing high sequence conservation at the C-termini and more diversity at the N-termini. The C-termini of the *B. rapa*, *B. oleracea* and *S. irio* homologs, but not of the *A. lyrata* homolog, contain two adjacent cysteine residues. Although cysteine residues are often implicated in disulphide bond formation to enhance protein stability, the adjacent localization of these residues in EWR1 makes intramolecular disulphide bond formation unlikely. However, possibly the cysteine residues might be involved in EWR1 homodimerization.

The search for recognizable protein domains in EWR1 did not result in any significant hits in publicly available databases. The absence of a functional annotation and any known motif or domain in EWR1 complicates the prediction of EWR1 function. The presence of an N-terminal signal peptide, an overall net positive charged (+2), and a relatively high number of hydrophobic amino acids (41%) are typical features that are shared with many antimicrobial peptides (AMPs) [30], [31], [32]. AMPs are found in all living organisms [32], [33]. In plants, six different AMPs families have been described, comprising thionins, defensins, lipid transfer proteins, knottins, heveins, and snakins, of which defensins are the largest group and best characterised [30], [31], [32], [33]. In Arabidopsis, 825 small cysteine-rich proteins with typical features of antimicrobial peptides have been predicted [34]. Several lines of evidence indicate that AMPs play role in plant defence against viral, bacterial and fungal pathogens [30], [31], [32], [33], [35]. AMPs are expressed in plants both constitutively and in response to pathogen attack [30], [36]. It has been shown that constitutive over-expression of AMPs increases plant defence against bacterial and fungal pathogens. For instance, the constitutive over-expression of the alfalfa defensin (*alfAFP*) in potato provides resistance against *V. dahliae* [37]. Similarly, constitutive expression of the radish defensin in tobaco and tomato, provides resistance against *Alternaria longipes* and *Alternaria solani*, respectively [30]. An *in vitro* EWR1 antimicrobial activity assay should answer whether EWR1 function as an AMP. So far, attempts for heterologous EWR1 protein production using *Escherichia coli* or the yeast *Pichia pastoris* system have not been successful.

Vascular wilt symptoms such as wilting, stunting, chlorosis and leaf defoliation are similar to those symptoms caused by drought stress. Indeed, the physical presence of vascular wilt pathogens in the xylem vessels, enzymes secreted by the fungus or plant defence responses may interfere with water transport in the xylem [28], [38]. In potato, it has been shown that Verticillium resistant potato cultivars exhibit drought stress tolerance [39]. We observed that *EWR1* over-expressing Arabidopsis plants similarly show drought stress tolerance. Leaf morphology such as size, thickness and shape has direct implication on water loss through transpiration [40], [41]. *EWR1* over-expressing plants have a smaller leaf size; have thicker and curly leaves than wild-type plants, which all can contribute to the amount of water loss through transpiration. Determining the effect of *EWR1* over-expression on the number of open stomata and the amount of water loss through transpiration in *EWR1* over-expressing plants when compared to the wild-type may provide insight in how *EWR1* regulates drought stress resistance.

For years, it has been a major focus for plant breeders to identify effective and durable genetic resistance to a wide range of pathogens. However, most of the resistance genes identified so far are either race- or species-specific and thus provide resistance to a limited number of pathogens. Thus, to obtain effective and durable resistance, it requires the transfer of multiple resistant genes into a cultivar. Since most AMPs have both antibacterial and antifungal activities and can be used across eukaryotic kingdoms [30], [37], [42], [43], [44], [45], they can potentially be used for developing effective resistance in plants against a broad spectrum of pathogens. Here we identified an Arabidopsis gene, *EWR1*, possibly encoding an AMP, which is effective at least against three vascular wilt pathogens. Moreover, heterologous expression of *AtEWR1* and *BoEWR1* in *N. benthamiana*, a plant species that belongs to the Solanaceae, confers Verticillium wilt resistance, making *EWR1* a potential gene to control vascular wilt pathogens in Brassicaceae and non-Brassicaceae plant species.

## Materials and Methods

### Plant inoculations

Arabidopsis plants and the microbial pathogens *V. dahliae* (isolates JR2, Dvd S26), *V. albo-atrum* (isolate #5431), *F. oxysporum* f.sp. *raphani* (strain #815), *P. syringae* p.v. *tomato* (strain DC3000), and *R. solanacearum* (strain GMI1000 and RD-15) were cultivated and inoculated as reported previously [23].

### Determination of the activation-tag insertion site

The activation-tag insertion site in mutant A2 was determined using thermal asymmetric interlaced PCR (TAIL-PCR) [46]. The PCR was performed with a combination of nested primers [47] and 10-mer random primers [48]. The secondary and tertiary TAIL-PCRs were separated on 1.2% agarose gel, stained with ethidium bromide, and visualized using the ChemiDoc XRS system (Bio-Rad). Specific product, judged based on the size differences generated by the nested primers, was excised, cleaned using the QIAquick Gel Extraction Kit (QIAGEN), cloned into the pGEM-T Easy Vector (Invitrogen), and sequenced. Blastn search of the TAIR database using the PCR sequences was performed to identify the genomic insertion site. Based on the putative insertion site, the primer pair MPR15F and MPR15R were designed and used to amplify the flanking genomic region. By sequencing this region in the wild-type and the mutant A2, the exact insertion site was determined.

### *EWR1* over-expression

The *EWR1* CDS was amplified with the primer pair dMRP15-F1 and dMRP15-R1 that contain *Bam*HI and *Asc*I restriction sites, respectively, using *Pfu* DNA polymerase (Promega). The amplicon was cloned into the *Bam*HI-and *Asc*I-pre-digested binary vector pmk40, a variant of the vector pmog800 [8], [49]. The resulting *P35S*:*EWR1* vector construct was transformed into *A. tumefaciens* strain GV3101 and eventually in to WS and Col-0 Arabidopsis ecotypes using the floral dip technique [50].

### Cloning of *EWR1* homologs

Primer pair EVR1H-BrF0 and EVR1H-BrR1 was used to amplify *BoEWR1* from genomic DNA (gDNA) of *Brassica oleracea* (Brussels sprout). The PCR product was excised from the gel, cleaned (GE Healthcare) and cloned into the pGMET-easy vector (Promega) and sequenced. Based on the sequence alignment of the PCR sequence and the *B. rapa* sequence in the database, primer EVR1H-BrR3 was designed and used in combination with EVR1H-BrF0 to amplify the predicted full length CDS of *BoEWR1* from *B. oleracea* cDNA. As a control, the same primer combination was used to amplify *BoEWR1* from gDNA. The PCR fragments were sequenced to confirm the full length CDS. To generate an *BoEWR1* over-expression construct, the full length CDS of *BoEWR1* was amplified from cDNA using primer pair EVR1H-BaF1 and EVR1H-AsR1 containing *BamH*I and *Asc*I custom restriction sites, respectively, and cloned into *BamH*I and *Asc*I pre-digested binary vector pB7K40 [23]. Subsequently, the binary vector construct was transformed into *A. tumefaciens* (strain GV3101) and eventually into Arabidopsis ecotypes WS and Col-0.

### Expression of *EWR1* homologs in *N. benthamiana*

In order to test whether expression of *AtEWR1* and *BoEWR1* results in Verticillium wilt resistance in non-Brassicaceae plants as well, the binary vectors containing *AtEWR1* or *BoEWR1* (described above) were transformed into *N. benthamiana*, a Solanaceae family member, following a standard *N. benthamiana* transformation protocol [51]. *AtEWR1* and *BoEWR1* transformed calli were selected on Kanamycin (50 µg/ml) and ammonium glufosinate (Basta  = 25 µg/ml) plates, respectively. After root generation, about 20 independent transformants per constructs were transferred to a soil for seed production. Subsequently, transformants were tested with PCR for transgene using Kanamycin and Basta specific primers, respectively. T2 seeds were harvested and three PCR-positive lines were selected and used in the preliminary Verticillium assay. Before inoculation, both the wild-type, *AtEWR1*, and *BoEWR1* over-expressing *N. benthamiana* plants were grown for four weeks in a greenhouse. Subsequently, plants were carefully uprooted, the roots were rinsed in water, and eventually inoculation was performed by root-dipping method as described for tomato and Arabidopsis [9], [23].

### Pathogen quantification *in planta*

Real-time PCR was used for quantification of pathogen colonization *in planta* using an ABI7300 PCR machine (Applied Biosystems) in combination with the qPCR Core kit for SYBR Green I (Eurogentec, Maastricht, The Netherlands) and analyzed using the 7300 System SDS software (Applied Biosystems). Unless described otherwise, the primer pair AtRub-F4 and AtRub-R4 targeting the gene encoding the large subunit of *RuBisCo* was used as endogenous control. *Verticillium* and *R. solanacearum* colonization was assessed as previously described [13], [23].

### Expression analysis

Both reverse transcription PCR and real-time PCR were used to analyze gene expression. Unless described otherwise, the primer pair Act2-F2 and Act2-R2 targeting the Arabidopsis *Actin 2* gene was used as endogenous control. A list of primers used in this study and their targets is presented in Table S1 in File S1. The real-time PCR conditions consisted of 2 min incubation at 50°C and 10 min at 95°C followed by 40 cycles of 95°C for 15 sec. and 60°C for 1 min.

## Supporting Information

File S1 Figure S1. Expression analysis of genes flanking the insertion site in the A2 mutant when compared to wild-type (WS) plants in absence of pathogen inoculation. The gene encoding EWR1 is boxed (*At3g13437*). Reactions to amplify the *Actin 2* gene and a non-template control (NTC) were included as controls. Figure S2. Relative quantification (RQ) of *EWR1* transcription. (A) Relative quantification (RQ) of *EWR1* transcription in wild-type (WS) and activation-tagged mutants (A1–A4). Bars represent averages with standard deviation of three biological replicates. (B) Relative quantification (RQ) of *EWR1* transcription in wild-type (Col-0) plants, two independent EWR1 over-expressing plants (*EWR1-1* and *EWR1-2*), the A2 mutant, and of the *EWR1* knock-out line (*ewr1*). Bars represent averages with standard deviation of three biological replicates. Figure S3. *EWR1* over-expressing Arabidopsis plants are resistant to *V. dahliae*. (A) Typical symptoms of *V. dahliae* on the wild-type (WS) and three independent *EWR1* over-expressing lines in WS background (AtEWR1-4, AtEWR1-5, and AtEWR1-6) at 21 days post inoculation (dpi). Representative of three experimental replicates is shown. (B) Disease severity score for the wild-type (WS) and three independent EWR1 over-expressing lines in WS background (AtEWR1-4, AtEWR1-5, and AtEWR1-6) at 14 (white bar) and 21 (grey bar) dpi. The total number of rosette leaves and number of rosette leaves that showed *Verticillium* symptoms was counted at least from eight plants and percentage of the disease leaves were calculated as an indication of disease severity. The bars represent averages of three independent experiments with standard deviation and asterisks indicate significance differences (Dunnett t-test at P = 0.05). Figure S4. *AtEWR1* over-expression alters Arabidopsis leaf morphology when compared to the wild-type (Col-0). Figure S5. Transcriptional regulation of *EWR1* gene during *Verticillium* infection. Relative quantification of *EWR1* transcription levels in the wild-type WS (white bar) and Col-0 (grey bar) plants at 0 (before inoculation), 4, 8, 12, and 17 days post *Verticillium* inoculation. The bars represent average and standard deviation of three technical replicates. Representative of three independent experimental replicates is shown. Figure S6. Relative quantification of *AtEWR1* transcription in the root and shoot of non-inoculated wild-type (WS) (white bar), the activation-tagged mutant A2 (light grey bar) and *AtEWR1* over-expressing line (EWR1-4) (dark grey bar). The EWR1 transcript level in the shoot of WS is set at one and used for calibration. A representative of two independent biological replications is shown and bar indicates average of three technical replicates and standard deviation. Figure S7. Relative quantification of *EWR1* transcript levels in *AtEWR1* (A) and *BoEWR1* (B) over-expressing *N. benthamiana* plants. The real-time PCR assays were normalized to the Arabidopsis actin transcript level as an internal control. The experiment was repeated at least three times with similar result and the bar indicates average of three technical replicates and standard deviation. Table S1: Primers used in this study.
(DOC)
