# Supplementary material for: The Brassicaceae-Specific EWR1 Gene Provides Resistance to Vascular Wilt Pathogens
Source: PLoS One. 2014 Feb 5;9(2):e88230. doi: 10.1371/journal.pone.0088230 (PMC3914955; doi:10.1371/journal.pone.0088230)
Supplement: File S1 — Figure S1. Expression analysis of genes flanking the insertion site in the A2 mutant when compared to wild-type (WS) plants in absence of pathogen inoculation. The gene encoding EWR1 is boxed (At3g13437). Reactions to amplify the Actin 2 gene and a non-template control (NTC) were included as controls. Figure S2. Relative quantification (RQ) of EWR1 transcription. (A) Relative quantification (RQ) of EWR1 transcription in wild-type (WS) and activation-tagged mutants (A1–A4). Bars represent averages with standard deviation of three biological replicates. (B) Relative quantification (RQ) of EWR1 transcription in wild-type (Col-0) plants, two independent EWR1 over-expressing plants (EWR1-1 and EWR1-2), the A2 mutant, and of the EWR1 knock-out line (ewr1). Bars represent averages with standard deviation of three biological replicates. Figure S3. EWR1 over-expressing Arabidopsis plants are resistant to V. dahliae. (A) Typical symptoms of V. dahliae on the wild-type (WS) and three independent EWR1 over-expressing lines in WS background (AtEWR1-4, AtEWR1-5, and AtEWR1-6) at 21 days post inoculation (dpi). Representative of three experimental replicates is shown. (B) Disease severity score for the wild-type (WS) and three independent EWR1 over-expressing lines in WS background (AtEWR1-4, AtEWR1-5, and AtEWR1-6) at 14 (white bar) and 21 (grey bar) dpi. The total number of rosette leaves and number of rosette leaves that showed Verticillium symptoms was counted at least from eight plants and percentage of the disease leaves were calculated as an indication of disease severity. The bars represent averages of three independent experiments with standard deviation and asterisks indicate significance differences (Dunnett t-test at P = 0.05). Figure S4. AtEWR1 over-expression alters Arabidopsis leaf morphology when compared to the wild-type (Col-0). Figure S5. Transcriptional regulation of EWR1 gene during Verticillium infection. Relative quantification of EWR1 transcription levels i [file pone.0088230.s001.doc]

**The novel *Arabidopsis thaliana* gene *EWR1* provides resistance to vascular wilt pathogens**

**Yadeta et al.**

**Supplemental Data**

**
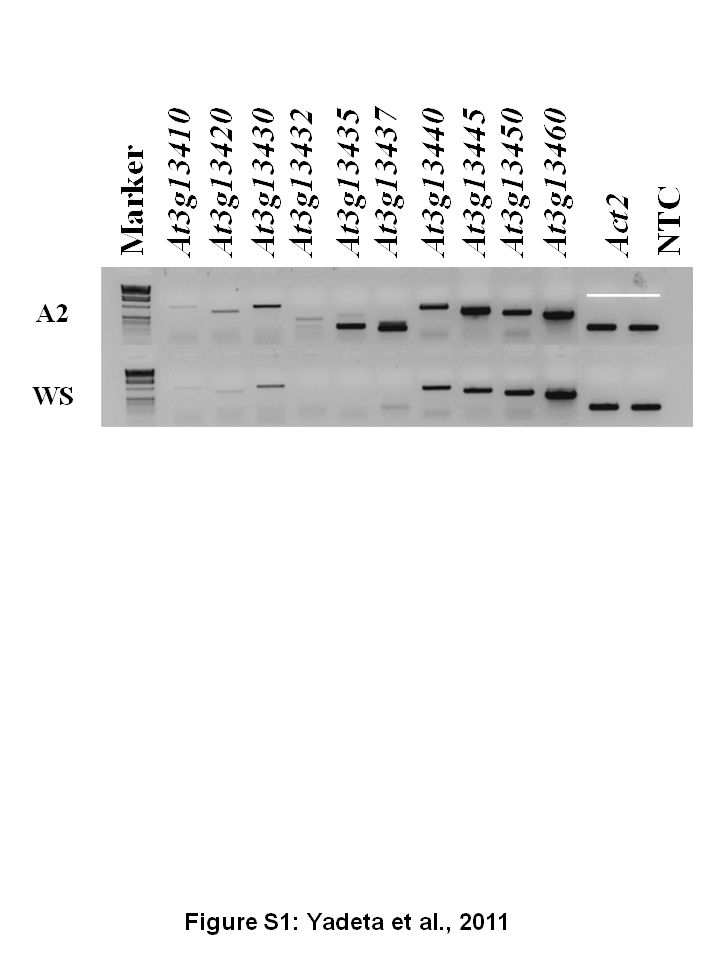
**

Figure S1. Expression analysis of genes flanking the insertion site in the A2 mutant when compared to wild-type (WS) plants in absence of pathogen inoculation. The gene encoding EWR1 is boxed (*At3g13437*). Reactions to amplify the *Actin 2* gene and a non-template control (NTC) were included as controls.

**
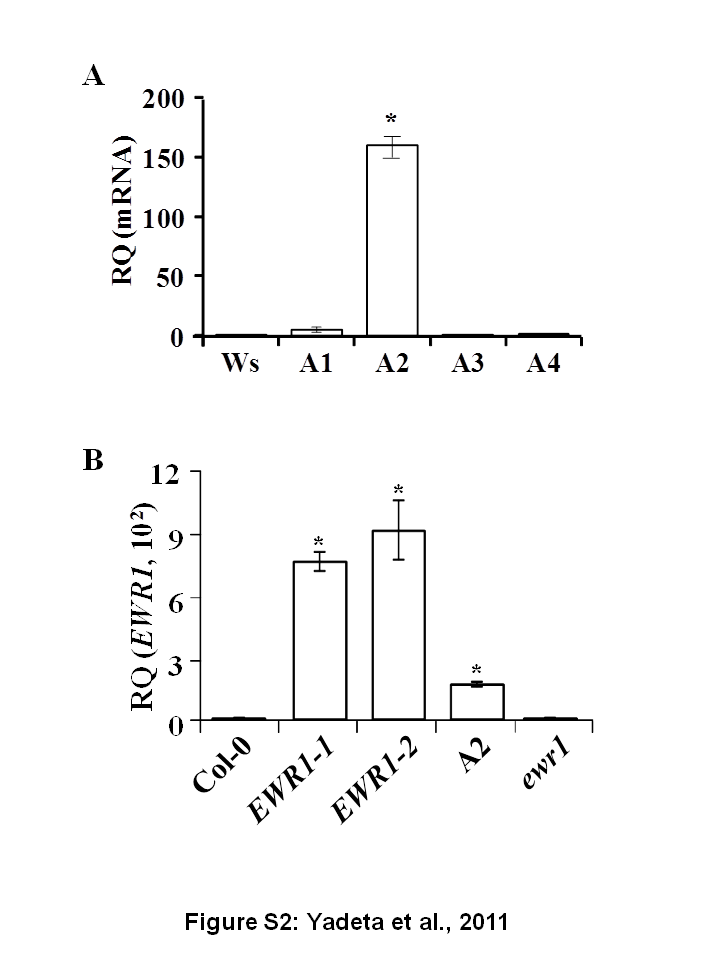

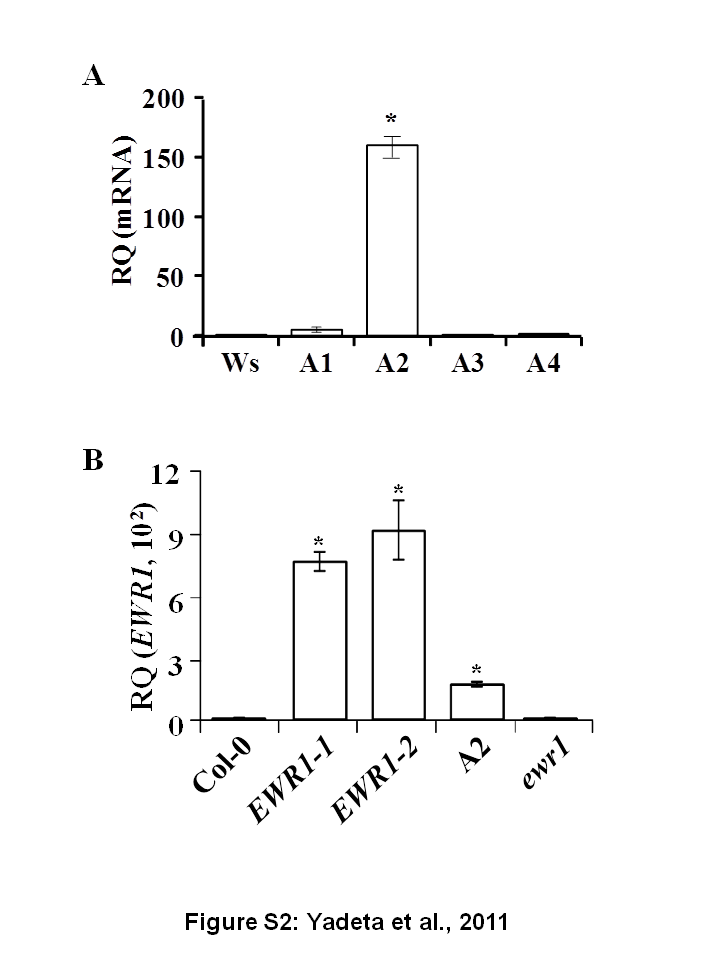
**

Figure S2. Relative quantification (RQ) of *EWR1* transcription. (A) Relative quantification (RQ) of *EWR1* transcription in wild-type (WS) and activation-tagged mutants (A1-A4). Bars represent averages with standard deviation of three biological replicates. (B) Relative quantification (RQ) of *EWR1* transcription in wild-type (Col-0) plants, two independent *EWR1* over-expressing plants (EWR1-1 and EWR1-2), the A2 mutant, and of the *EWR1* knock-out line (*ewr1*). Bars represent averages with standard deviation of three biological replicates.

**
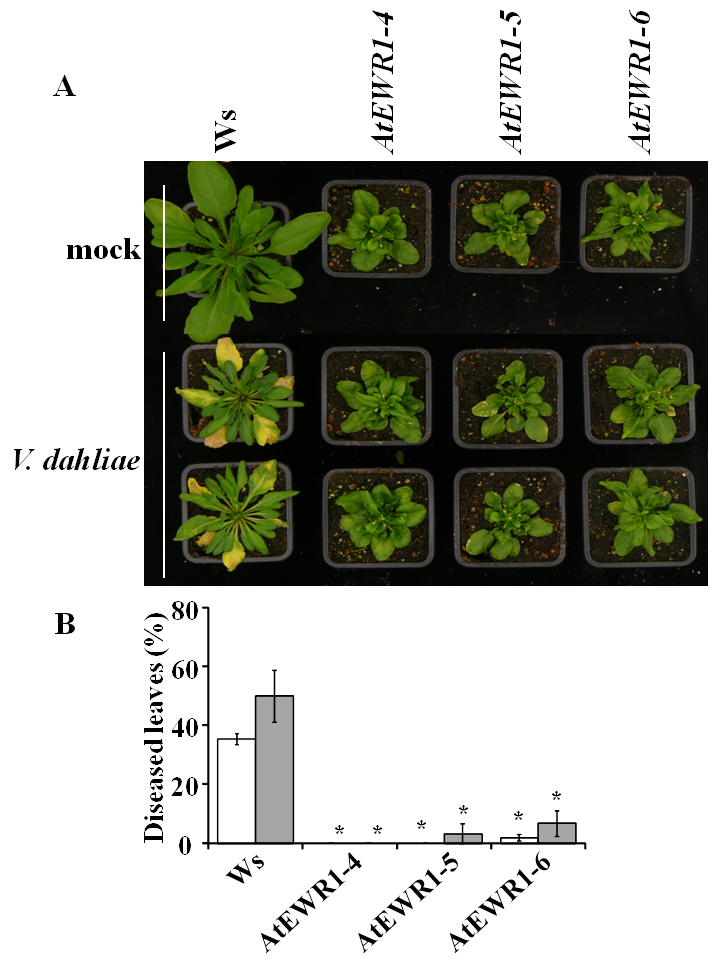
**

Figure S3. *EWR1* over-expressing Arabidopsis plants are resistant to *V. dahliae*. (A) Typical symptoms of *V. dahliae* on the wild-type (WS) and three independent *EWR1* over-expressing lines in WS background (AtEWR1-4, AtEWR1-5, and AtEWR1-6) at 21 days post inoculation (dpi). Representative of three experimental replicates is shown. (B) Disease severity score for the wild-type (WS) and three independent *EWR1* over-expressing lines in WS background (AtEWR1-4, AtEWR1-5, and AtEWR1-6) at 14 (white bar) and 21 (grey bar) dpi. The total number of rosette leaves and number of rosette leaves that showed *Verticillium* symptoms was counted at least from eight plants and percentage of the disease leaves were calculated as an indication of disease severity. The bars represent averages of three independent experiments with standard deviation and asterisks indicate significance differences (Dunnett t-test at *P=0.05*).

**
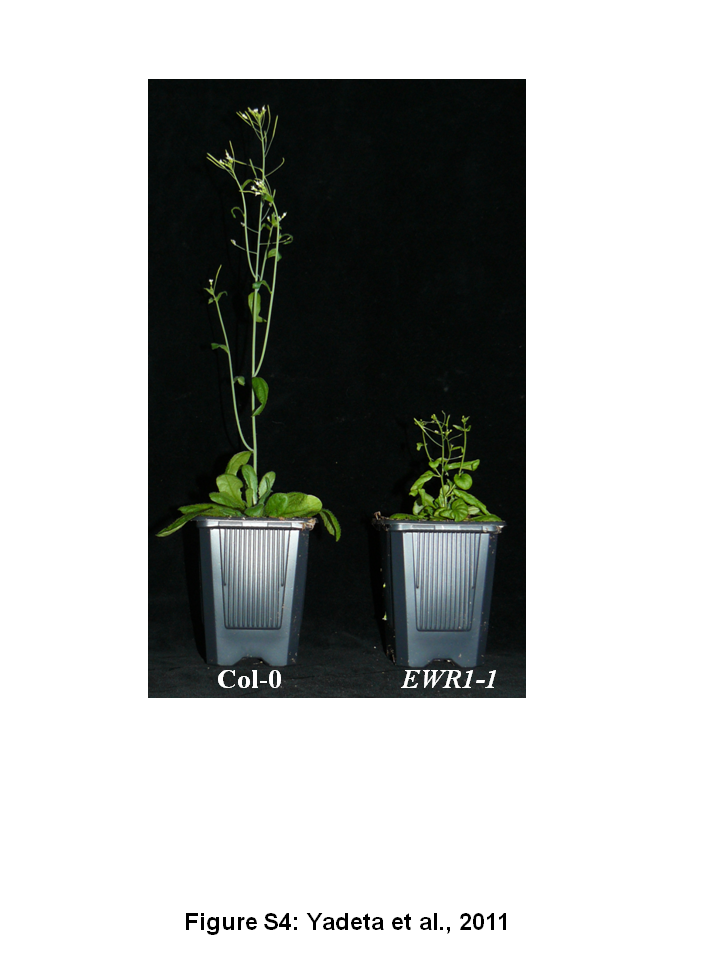
**

Figure S4. *AtEWR1* over-expression alters Arabidopsis leaf morphology when compared to the wild-type (Col-0).

**
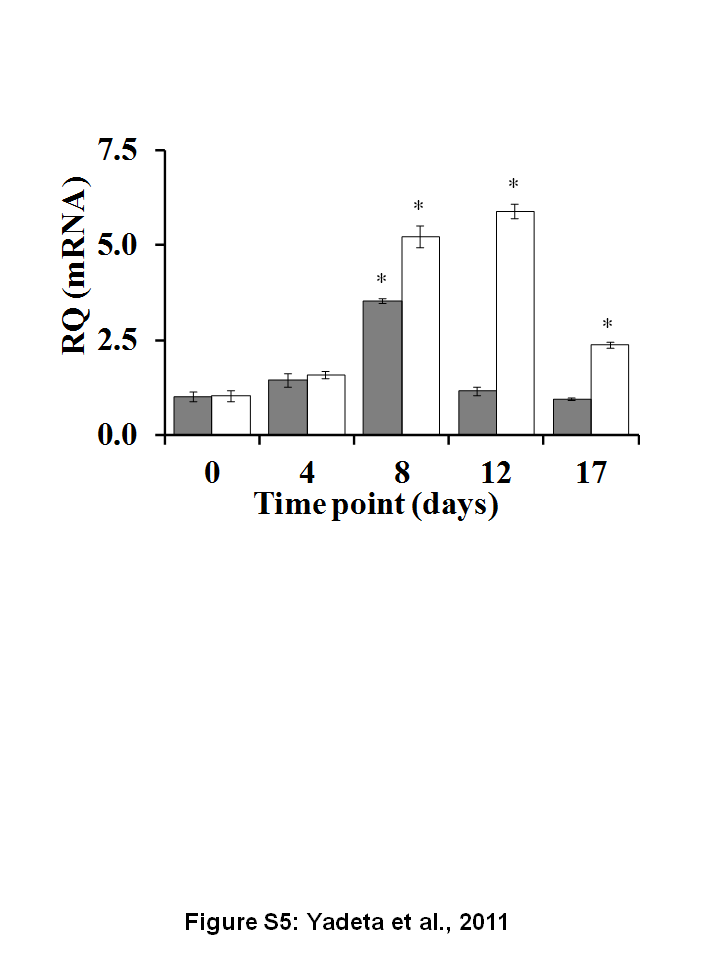
**

Figure S5. Transcriptional regulation of *EWR1* gene during *Verticillium* infection. Relative quantification of *EWR1* transcription levels in the wild-type WS (white bar) and Col-0 (grey bar) plants at 0 (before inoculation), 4, 8, 12, and 17 days post *Verticillium* inoculation. The bars represent average and standard deviation of three technical replicates. Representative of three independent experimental replicates is shown.

**
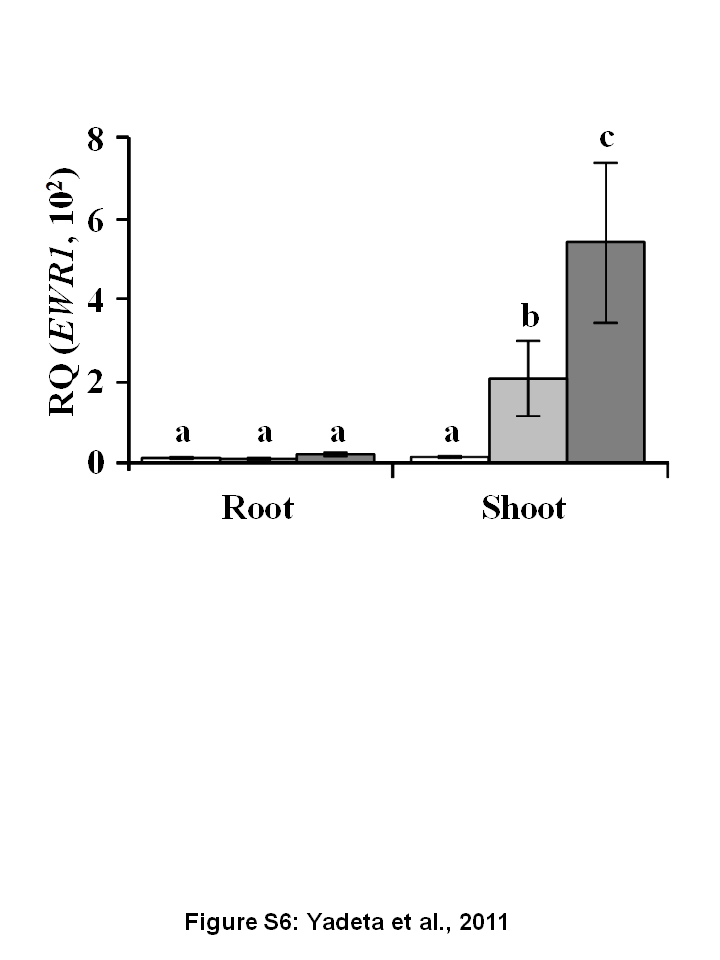
**

Figure S6. Relative quantification of *AtEWR1* transcription in the root and shoot of non-inoculated wild-type (WS) (white bar), the activation-tagged mutant A2 (light grey bar) and *AtEWR1* over-expressing line (EWR1-4) (dark grey bar). The *EWR1* transcript level in the shoot of WS is set at one and used for calibration. A representative of two independent biological replications is shown and bar indicates average of three technical replicates and standard deviation.

**
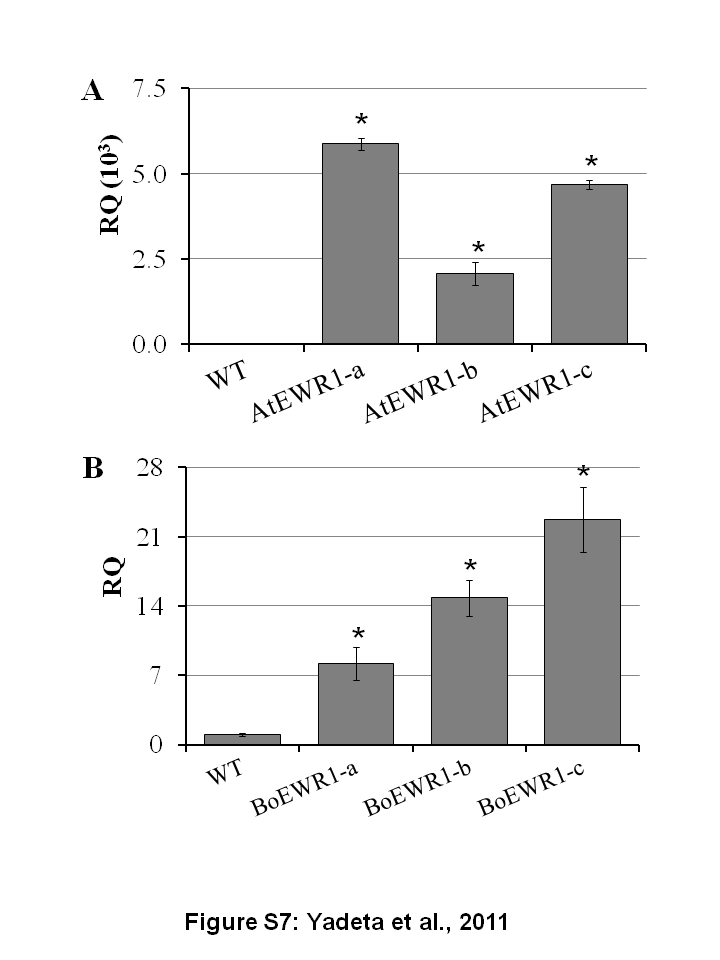

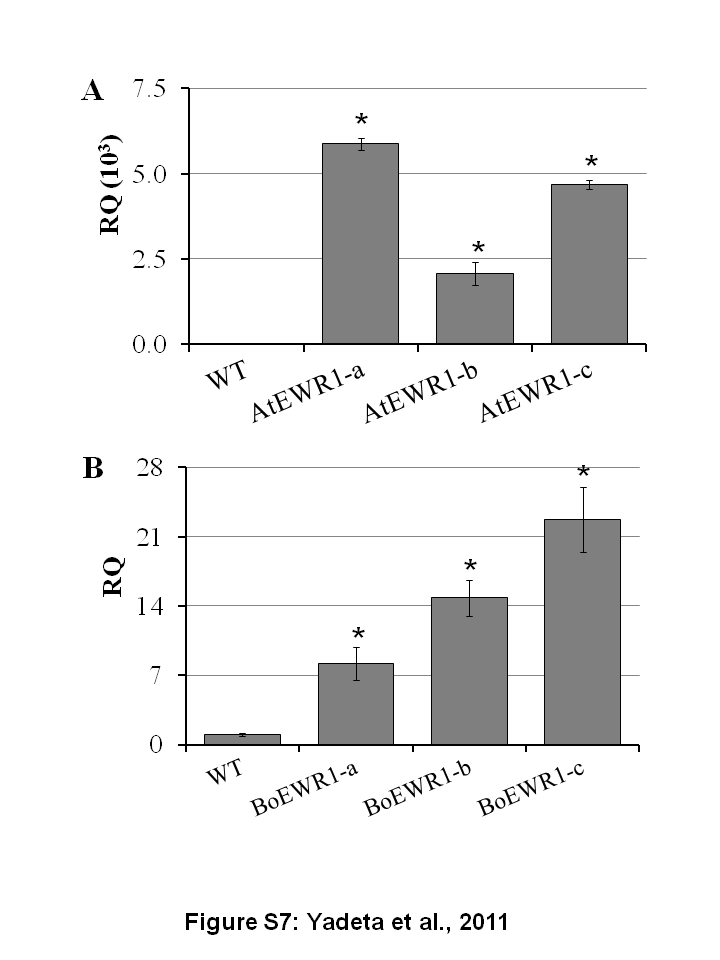
**

Figure S7. Relative quantification of *EWR1* transcript levels in *AtEWR1* (A) and *BoEWR1* (B) over-expressing *N. benthamiana* plants. The real-time PCR assays were normalized to the Arabidopsis actin transcript level as an internal control. The experiment was repeated at least three times with similar result and the bar indicates average of three technical replicates and standard deviation.

Table S1: Primers used in this study

| Primer code | Sequence (5' to 3') | Purpose |
| --- | --- | --- |
| MPR15F | ACCTTGTCTTTTGTATTCACTG | Confirmation of activation tag insertion site |
| MPR15R | AAGTTTGGAACGAGGCAG | Confirmation of activation tag insertion site |
| MPR15-F1 | GGAGTTTTGTACTTTGCGACG | Confirmation of activation tag insertion site |
| MPR15-R1 | AGTTTGGAACGAGGCAGC | Confirmation of activation tag insertion site |
| dMRP15-2F1 | GCATCACATTTTCCAATTCGAC | *AtEWR1* expression analysis (RT-PCR) |
| dMRP15-2R1 | CATTGCAACAAATCCAGC | *AtEWR1* expression analysis (RT-PCR) |
| dMRP15-F1 | **GGATCC**ATGAGTCTCAAGTTCATTC | *AtEWR1* over-expression construct (*BamH*I) |
| dMRP15-R1 | **GGCGCGCC**TTAATCATTGCAACAAATCC | *AtEWR1* over-expression construct (*Asc*I) |
| ITS1-F | AAAGTTTTAATGGTTCGCTAAGA | *Verticillium* quantification (Ellendorf et al., 2009) |
| St-Ve1-R | CTTGGTCATTTAGAGGAAGTAA | *Verticillium* quantification (Ellendorf et al., 2009) |
| AtRub-F4 | GCAAGTGTTGGGTTCAAAGCTGG | *Verticillium* quantification (Yadeta et al., 2011) |
| AtRub-R4 | AACGGGCTCGATGTGGTAGC | *Verticillium* quantification (Yadeta et al., 2011) |
| EVR1-F1 | GTATCACACCAACTGTAATGAGAACG | T-DNA insertion check |
| EVR1-R1 | TTAATCATTGCAACAAATCCAG | T-DNA insertion check |
| EVR1-eF1 | **CGGTATGAATTCcatcatcatcatcatcatcccgactacaaggacgacgatgacaag**  ACCGTCGTTCCTTTTTCC | AtEWR1 protein expression (His6-FLAG-AtEWR1^-Sp^ ) |
| EVR1-nR1 | CGTCTA**GCGGCCGC**TTAATCATTGCAACAAATCC | *AtEWR1* protein expression in *P. pastoris* (His6-FLAG-AtEWR1-^Sp^ ) |

Table S1: Continued...

| Primer code | Sequence (5' to 3') | Purpose |
| --- | --- | --- |
| EVR1H-BrF0 | ATGAGTCTCAAGTTCATT | Cloning *BoEWR1* |
| EVR1H-BrR1 | CAGAGCTTCTTTTAATCATTGC | Cloning *BoEWR1* |
| EVR1H-BrR3 | TTAATCATTGCAGCAATT | Cloning *BoEWR1* |
| EVR1H-BaF1 | **GCAGGATCC**ATGAGTCTCAAGTTCATT | Making BoEWR1 over-expression construct |
| EVR1H-AsR1 | **ACTGGCGCGCC**TTAATCATTGCAGCAATT | Making BoEWR1 over-expression construct |
| Act2-F2 | TAACTCTCCCGCTATGTATGTCGC | Arabidopsis act2 gene (Endogenous control ) |
| Act2-R2 | GAGAGAAACCCTCGTAGATTGGC | Arabidopsis act2 gene (Endogenous control ) |
| dMRP15-1F1 | GAATTGGAAGTTGGTTTTGC | Expression analysis |
| dMRP15-1R1 | AGAAATGATCTTCGGTGG | Expression analysis |
| dMRP15-2F1 | GCATCACATTTTCCAATTCGAC | Expression analysis |
| dMRP15-2R1 | CATTGCAACAAATCCAGC | Expression analysis |
| dMRP15-3F1 | AGAGAGTAATCCAATGGACC | Expression analysis |
| dMRP15-3R1 | GATGTCTCTTTGTCCTGG | Expression analysis |
| dMRP15-4F1 | GATTGGAAGGGAGTAATCC | Expression analysis |
| dMRP15-4R1 | TCTGAATTCCGAGAGCAC | Expression analysis |
| uMRP15-1F1 | GTTCTGTTTGATTGCTTCCC | Expression analysis |
| uMRP15-1R1 | CTGAATTTGGACTTGCGG | Expression analysis |
| uMRP15-2F1 | CATCAGAGACTAGCTACTGG | Expression analysis |
| uMRP15-2R1 | GTTCGAACTTGAGTCTGG | Expression analysis |
| uMRP15-3F1 | GCTTTGTGTTTCGTTACG | Expression analysis |
| uMRP15-3R1 | AAGACCTGTGTTGCATTG | Expression analysis |
| uMRP15-4F1 | GTGTTTCTATCTGTGGCC | Expression analysis |
| uMRP15-4R1 | GAATCTTGAGGAGTCTCG | Expression analysis |

Table S1: Continued...

| Primer code | Sequence (5' to 3') | Purpose |
| --- | --- | --- |
| AtEVR1-F | GCATCACATTTTCCAATTCGAC | Expression of *AtEWR1* in *N. benthamiana* |
| AtEVR1-R | CATTGCAACAAATCCAGC | Expression of *AtEWR1* in *N. benthamiana* |
| BoEVR1-F | ATGAGTCTCAAGTTCATT | Expression of *AtEWR1* in *N. benthamiana* |
| BoEVR1-R | CAGAGCTTCTTTTAATCATTGC | Expression of *AtEWR1* in *N. benthamiana* |
| NbActin-F | CCAGGTATTGCCGATAGAATG | *N.benthamiana* Actin gene (endogenous control) |
| NbActin-R | GAGGGAAGCCAAGATAGAGC | *N. benthamiana* Actin gene (endogenous control) |
